# Adsorption Evaluation for the Removal of Nickel, Mercury, and Barium Ions from Single-Component and Mixtures of Aqueous Solutions by Using an Optimized Biobased Chitosan Derivative

**DOI:** 10.3390/polym13020232

**Published:** 2021-01-11

**Authors:** Efstathios V. Liakos, Mariza Mone, Dimitra A. Lambropoulou, Dimitrios N. Bikiaris, George Z. Kyzas

**Affiliations:** 1 Department of Chemistry, International Hellenic University, 65404 Kavala, Greece; stathilas@gmail.com; 2 Laboratory of Polymer Chemistry and Technology, Department of Chemistry, Aristotle University of Thessaloniki, 54124 Thessaloniki, Greece; mariza@chalmers.se (M.M.); dbic@chem.auth.gr (D.N.B.); 3 Laboratory of Environmental Pollution Control, Department of Chemistry, Aristotle University of Thessaloniki, 54124 Thessaloniki, Greece; dlambro@chem.auth.gr

**Keywords:** chitosan, 5-hydroxymethyl-furfural, derivative, biomaterials, adsorption, nickel, mercury, barium, wastewaters

## Abstract

In this experimental study, the use of 5-hydroxymethyl-furfural (HMF) organic compound as a grafting agent to chitosan natural polymer (CS) was examined. One optimized chitosan derivative was synthesized, and then tested (CS-HMF), in order to uptake nickel, mercury, and barium metal ions from single- and triple-component (multi-component) aqueous solutions. The characterization of the material before and after the metal uptake was achieved by scanning electron microscopy (SEM). The ability of the adsorption of CS-HMF was tested at pH = 6. The adjusting of temperature from 25 to 65 °C caused the increase in the adsorption capacity. The equilibrium data were fitted to the models of Langmuir and Freundlich, while the data from kinetic experiments were fitted to pseudo-1st and pseudo-2nd order models. The best fitting was achieved for the Langmuir model (higher R^2^). The adsorption capacity for nickel, mercury, and barium removal at 25 °C (single component) was 147, 107, and 64 (mg/g), respectively. However, the total adsorption capacity for the multi-component was 204 mg/g. A thermodynamic study was also done, and the values of ΔG^0^, ΔH^0^, and ΔS^0^ were evaluated.

## 1. Introduction

Water pollution with toxic metals is a serious public and environmental problem in nowadays, on a worldwide scale. Furthermore, water pollution is a priority for most sectors of industries, due to the enhanced environmental concern [1,2,3,4,5]. Heavy metals tend to accumulate in receiving water bodies [6] and flora [7], because they are not self-degradable, with result to cause various disorders and diseases. Consequently, their presence particularly in water should be controlled. Special attention must be given in three heavy metal ions like mercury, nickel, and barium.

Mercury metal ions (Hg^2+^) are very toxic, with a significant harmful factor to the environment [6]. The main sources for the discharge of Hg^2+^ into aqueous system are naturally caused forest fires, paint and chloralkali, fossil fuel burning, battery production industries, etc., [8,9]. The Hg^2+^ uptake from human can cause dermatitis, erosion to skin, muscles, eyes, kidney damage, impairment of pulmonary function, and neurological and renal disturbances [10]. In addition, nickel metal ions (Ni^2+^) can cause serious pollution of water, and this type of aqueous pollution is produced mainly from electronics, electroplating, and metal cleaning industries [11]. As it is well known, Ni^2+^ has high toxicity [7] and carcinogenicity, even at low concentrations [12], and in the case where it is discharged into wastewaters of industries can be a serious threat to receiving water bodies [7]. In the base of barium ions (Ba^2+^), it is worth to note that barium element is the 14th most abundant element in the crust of earth. Consequently, barium concentrations in water, soil, and air, due to industries human activities, may be increased, on many locations, when compared with naturally occurring concentrations [13]. Ba^2+^ ions are radionuclides with extremely high toxicity and can be found in relatively large concentrations in liquid radioactive effluents produced from reprocessing plants. Even small amounts of Ba^2+^ may cause heart damage, heart rhythm changes, swelling of brains and liver, kidney damage, changes in nerve reflexes, stomach irritation, increased blood pressure, and muscle weakness [14,15].

The main methods for the metal ions uptake from industrial wastewaters are reverse osmosis, solvent extraction, ion exchange, chemical precipitation, membrane filtration, electrolytic methods, and adsorption [16,17,18]. However, these techniques have high operational cost and in the case of trace level concentrations may be ineffective for the adsorption of some types of toxic metal ions [16,19,20,21,22,23,24,25,26,27,28,29,30,31,32,33,34]. Therefore, a candidate facing the heavy metal ions pollutions is the use of adsorption technology [35,36,37,38,39], and specifically by using chitosan as an adsorbent. Chitosan is a biopolymer that is widely used for the adsorption of heavy metal ions from wastewaters, even at low initial concentrations [40,41]. It is a nitrogenous polysaccharide mainly with amino groups, which is generated in large amounts from chitin via *N*-deacetylation. Chitosan, due to its physical properties as biodegradability, nontoxicity, low cost, biocompatibility, macromolecular structure [42], anti-bacterial properties [11], etc., can be used (apart from adsorption) in many fields, including the food industry, biotechnologies, membranes, medicine, cosmetics, etc., but also its adsorption capacity [42].

In this study, an environmentally friendly adsorbent was synthesized in order to uptake heavy metal ions from aqueous solutions. As model pollutants, Ni^2+^, Hg^2+^, and Ba^2+^ were selected. The grafted chitosan derivative was produced by grafting 5-hydroxymethyl-furfural (HMF) into chitosan. It must be noted that this adsorbent material was applied based on our previous work [43] in which we tested as series of CS-HMF materials for adsorption of copper and cadmium ions. However, in the latter study, the CS-HMF was synthesized with various molecular ratios (CS/HMF of 1:1 (CS-HMF1), 2:1 (CS-HMF2), and 10:1 mol/mol (CS-HMF3)). The novelty of this work is that we used the optimum adsorbent material found (10:1 mol/mol (CS-HMF3)), and we have now tested three different heavy metal ions (Ni^2+^, Hg^2+^, and Ba^2+^), not only for single-component solutions (as the majority of papers), but also for mixture. The experiments of adsorption evaluation of CS-HMF were carried out with single- and multi(triple)-component aqueous solutions. The evaluation of adsorption was multi-parametric based on the optimum pH, temperature, and initial ion concentration of metal ions removal. The equations of Langmuir and Freundlich were fitted to the experimental data in the equilibrium phase. The experimental kinetic data were applied to the pseudo-1st and pseudo-2nd order equations to evaluate the kinetics during the process of adsorption.

## 2. Materials and Methods

### 2.1. Materials

All chemical reagents that used in the experimental step, for the synthesis of biobased adsorbent were purchased by Sigma-Aldrich (Berlin Germany). CS (>75% deacetylated, high molecular weight, 310–375 kDa), 5-hydroxymethylfurfural (HMF), sodium tripolyphosphate (98%), and Glutaraldehyde (50% *v*/*v*) were used as chemical reagents for the preparation of synthetic process. Some other reagents as K_2_HPO_4_·3H_2_O, Na_2_HPO_4_, NaCl, KCI, and HCl were also used. To synthesize the stock solutions of model pollutants (single- or multi-component aqueous solutions), the salts used (Ni(NO_3_)_2_·6H_2_O, Hg(NO_3_)_2_·H_2_O, BaCl_2_·2H_2_O) were also purchased from the same company.

### 2.2. Synthesis of CS-HMF Adsorbent

The synthetic preparation of CS-HMF was presented in our previous study [44]. In sum, the synthesis of CS-HMF was carried out by using one molecular ratio, more specifically 10:1 mol/mol (CS/HMF). In addition, on the following process is presented briefly the synthetic route of CS-HMF. The synthetic process starts with the dissolving of CS (10 g) in acetic acid solution (2% (*v*/*v*)), via stirring for overnight. Then, HMF (3.7 g) were dissolved in ethanol (50 mL) and afterward inserted in CS solution. The reaction process was achieved under magnetic stirring at 60 °C for 4 h, and after the aforementioned chronic period was inserted drop wised NABH_4_ (during the reaction). Furthermore, the obtained bioadsorbent material was lyophilized and purified using Soxhlet extraction. The process of Soxhlet extraction lasted for 24 h, and the solution that was used for the lyophilization and purification process was acetone. The process of cross-linking of CS-HMF was achieved using GLA and TPP solution, and this derivative was used only for studies of swelling. More specifically, CS-HMF (1 g) was inserted in aqueous solution and dissolved in the presence of acetic acid (50 mL, 4% (*v*/*v*)). Then, GLA solution was inserted (1% (*v*/*v*)) to TPP (1% (*v*/*v*)) solution (pH 6). Then, the resulting solution of CS was poured into the aforementioned synthesized coagulant solution for the process of gelation and stirred for overnight. Finally, the resulting solution lyophilized in order to obtain the final biobased adsorbent derivative (CS-HMF).

### 2.3. Characterization Techniques

The surface morphology of the synthesized grafted chitosan was evaluated by SEM images by using a Jeol JSM-6390 LV, Japan. The voltage of electron acceleration was 15.00 kV, while the step of scanning was applied in situ on the dry powder of CS-HMF derivative. The FTIR spectra were obtained, using a Perkins Elmer FT-IR/NIR spectrometer Frontier (USA), after 32 scans (400–4000 cm^−1^) at a resolution of 4 cm^−1^ and baseline correction (normalization to 1).

### 2.4. Adsorption Process

The design of the adsorption process can be divided into 3 main subsections, by testing the effect of some significant parameters. Initially, for the experiments of adsorption, in the case of single-composite aqueous solutions, stock aqueous solutions of Ni^2+^, Hg^2+^, and Ba^2+^ were synthesized in volumetric flasks by weighting a specific mass of salt (1000 mg/L), and then by filling with deionized water. It must be noted that the process of heavy metals ions adsorption was achieved via agitation of conical flasks by using a thermostatically controlled shaking bath. After the finish of process of heavy metal ions adsorption, the evaluation of remaining (effluent) concentration of ions, in single- and triple-component aqueous solutions, was achieved via atomic absorption spectroscopy (AAS) (Perkin-Elmer Analyst 400 with Flow Injection System-FIAS 100).

#### 2.4.1. pH Effect

In order to obtain the experimental results of adsorption process, during the effect of pH, an aqueous solution of metal ions (Ni^2+^, Hg^2+^, Ba^2+^) was prepared with adjusted initial ion concentration (C_0_ = 100 mg/L), where the volume of deionized water was constant for all cases (20 mL). The adjustment of agitation rate was at 160 rpm for 24 h (T = 25 °C). The appropriate mass (0.02 g) of chitosan derivative was added to the flasks, while the pH solution was adjusted at 2, 3, 4, 5, 6 with additions of HCI (0.01 mol/L) or NaOH (0.01 mol/L). To avoid precipitation phenomena of heavy metal ions (form of hydroxides) [45], the adsorption experiments were not carried out at pH > 6. The remaining concentrations of ion (C_e_), in the aqueous solution after the process of adsorption, were evaluated with AAS, and the uptake of metallic ions was determined as follows (Equation (1)):

(1) $$R=\frac{\left(C_{0}-C_{e}\right)}{C_{0}}\cdot 100\%$$

#### 2.4.2. Contact Time Effect

The experiments of kinetics were carried out in ion solutions of C_0_ = 100 mg/L (Ni^2+^, Hg^2+^, Ba^2+^), where the volume of deionized water was constant for all cases (20 mL). The adjustment of agitation rate was maintained at 160 rpm for 24 h (T = 25 °C). The appropriate mass (0.02 g) of chitosan derivative was inserted into flasks, while the pH adjusted at 6 (optimum pH) result is attributed to the pH effect experiments that are discussed above. The residual concentrations of ions were analyzed at predefined time intervals (5 min–24 h). Moreover, the calculations of residual concentrations of ion (C_e_) in the liquid phase were achieved according to the above section “pH effect”. The experimental data fitting was achieved by using two widely known kinetic equations; (i) pseudo-1st order equation [46] (Equation (2)), and (ii) pseudo-2nd order equation [47,48,49] (Equation (3)):

(2) $$C_{t}\;=\;C_{0}-(C_{0}-C_{e})\left(1-e^{-k_{1}t}\right)$$

(3) $$C_{t}\;=\;C_{0}-(C_{0}-C_{e})\left(1-\frac{1}{1+k_{2}t}\right)$$

where C_t_ (mg/L), k_1_ (min^−1^), and k_2_ (g mg^−1^ min^−1^) are the concentration of ion at a specific time interval, and the kinetic constants derived from pseudo-1st and pseudo-2nd order equations, respectively.

#### 2.4.3. Isotherms 

The experimental design of equilibrium adsorption capacity for single-component aqueous solutions were carried out by adding C_0_ = 10.0–300.0 mg/L of each metal, where the volume of deionized water was constant for all cases (20 mL). The adjustment of agitation rate was maintained at 160 rpm, under different temperatures (25, 45, and 65 °C). The appropriate mass (0.02 g) of chitosan derivative was inserted into flasks, while the pH adjusted at 6 (optimum pH) result is attributed to the pH effect experiments that are discussed above. The optimum duration of process of adsorption (process/contact time) was derived from the experiments of kinetics. Below, the equation is depicted, which is widely applied for the calculation of equilibrium amount in the solid surface of particle (Q_e_) (Equation (4)):

(4) $$Q_{e}=\frac{\left(C_{0}-C_{e}\right)V}{m}$$

The isotherm equations of Langmuir [50] and Freundlich [51] ((Equations (5) and (6)), respectively) were performed for the simulation of equilibrium data derived from the experimental process of adsorption.

(5) $$Q_{e}\;=\;\frac{Q_{m}K_{L}C_{e}}{1+K_{L}C_{e}}$$

(6) $$Q_{e}\;=\;K_{F}C_{e}{}^{1/n}$$

where Q_m_ (mg/g), K_L_ (L/mg), K_F_ (mg^1−1/n^ L^1/n^ g^−1^), and n (dimensionless) are mentioned as the theoretical total adsorption capacity, the Langmuir equilibrium constant, the Freundlich constant, and the dimensionless Freundlich constant, respectively.

#### 2.4.4. Mixtures

The experimental design of equilibrium adsorption capacity for multi-component aqueous solutions were carried out by adding C_0_ = 10.0–300.0 mg/L of each metal simultaneously, where the volume of deionized water was constant for all cases (20 mL). The adjustment of agitation rate was maintained at 160 rpm (25 °C). The appropriate mass (0.02 g) of chitosan derivative was inserted into flasks, while the pH adjusted at 6 (optimum pH) result is attributed to the pH effect experiments that are discussed above. The remaining concentration of each ion (C_e_) in the aqueous solution after the process of adsorption was evaluated with AAS, as described above. The calculation of the total equilibrium amount in the solid surface of particle (Q_e_) (Equation (7)) is as follows:

(7) $$\begin{array}{l} Q_{e,tot}=\frac{\left(C_{0,tot}-C_{e,tot}\right)V}{m}\\ \mathrm{where}\\ C_{0,tot}=C_{0,Ni}+C_{0,Hg}+C_{0,Ba}\\ \mathrm{and}\\ C_{e,tot}=C_{e,Ni}+C_{e,Hg}+C_{e,Ba} \end{array}$$

## 3. Results and Discussion

The experimental findings discussion begins with the morphology and functional groups of CS-HMF bioadsorbent, before and after the adsorption of Ni^2+^, Hg^2+^, and Ba^2+^. Then, the evaluation of adsorption process comes from the obtained results derived from isotherms and kinetics.

### 3.1. SEM and FTIR

**Figure 1:**
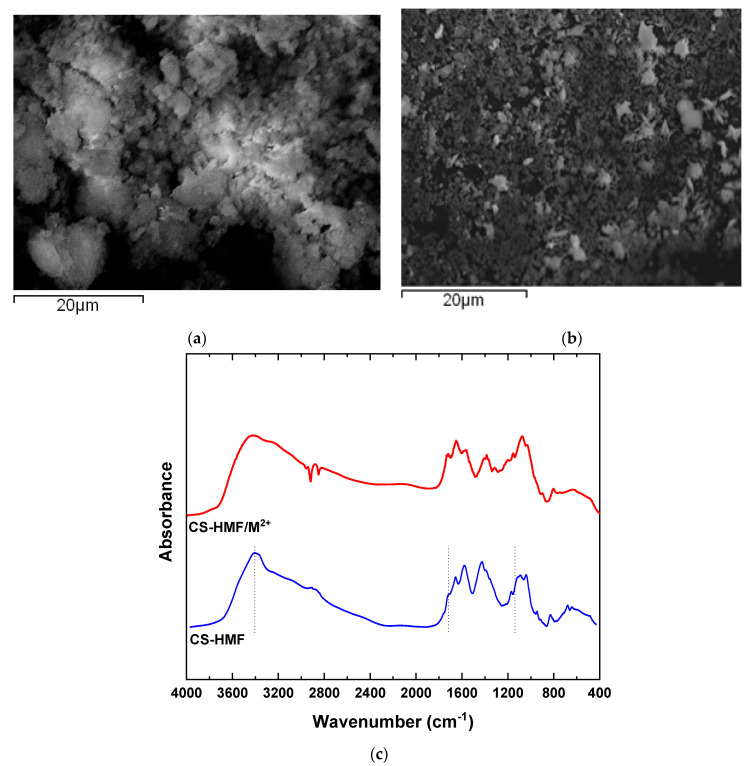
(**a**) SEM images of chitosan natural polymer 5-hydroxymethyl-furfural (CS-HMF) before metal ion removal; (**b**) SEM images of CS-HMF after metal ion removal (multi-component); (**c**) FTIR spectra of CS-HMF before and after metal ion removal.

The morphology of the CS-HMF derivative was evaluated with scanning electron microscopy. Figure 1a depicts the morphology of CS-HMF before the process of adsorption, while the morphology after metal ions adsorption is presented in Figure 1b. Moreover, in Figure 1c the obtained FTIR spectra before and after the metal ions adsorption is presented. According to Figure 1a, the CS-HMF adsorbent does not have a smooth surface morphology, resulting in irregular shape with a rough surface possibly due to the processes of cross-linking and grafting. In addition, Figure 1b depicts that the uptake of metal ions (Ni^2+^, Hg^2+^, Ba^2+^) caused by the smashing of the derivative, probably due to the extreme conditions of pH.

The spectra of CS-HMF presents the characteristic absorption bands at 1659 (Amide I), 1528 (Amide IΙ), and 1381 cm^−1^ (–CH_2_ bending). At 1156 cm^−1^, CS-HMF absorbs the vibration band of C–O–C, while at 1084 and 1028 cm^−1^ (skeletal vibrations involving the C–O stretching), peaks are related with the characteristic absorption bands of polysaccharides, such as chitosan. Moreover, a broad peak of hydroxyl groups of chitosan is obvious at 3399 cm^−1^. The spectra of pure HMF present a strong absorption band at 3360 cm^−1^ due to hydroxyl groups (–OH). The successful synthesis was verified with the presence of HMF peak at, e.g., absorption band 800 cm^−1^, which is attributed to the furanic ring. Parallel, their delocalization at lower wavenumbers, suggests the generation of hydrogen bonds between the amino- and carbonyl-groups of HMF.

After adsorption, the FTIR spectra show that the broad band before the process of adsorption at 3399 cm^−1^ was shifted to 3494 cm^−1^ after metal ions adsorption, indicating that the hydroxyl groups of HMF interact with the model pollutants. The band at 1704 cm^−1^ (–HC=O groups) is disappeared, indicating that this functional group is eliminated in water or interacts with heavy metal ions, because a new shifted peak is not presented. Moreover, the band at 1563 cm^−1^ (also assigned to the N−H bond) was decreased considerably in intensity and displaced to 1555 cm^−1^. According to all above FTIR findings, it was clear that a coordination complex was formed between CS-HMF and metals with the participation of the amino and hydroxyl functional groups of chitosan. In general, all interactions recorded, peaks and shifts, are attributed mainly due to the interactions between hydroxyl groups from HMF (as aforementioned) and free amino (carbonyl) functional groups of chitosan.

### 3.2. Adsorption Evaluation—Single-Component Solutions

#### 3.2.1. pH Effect

**Figure 2:**
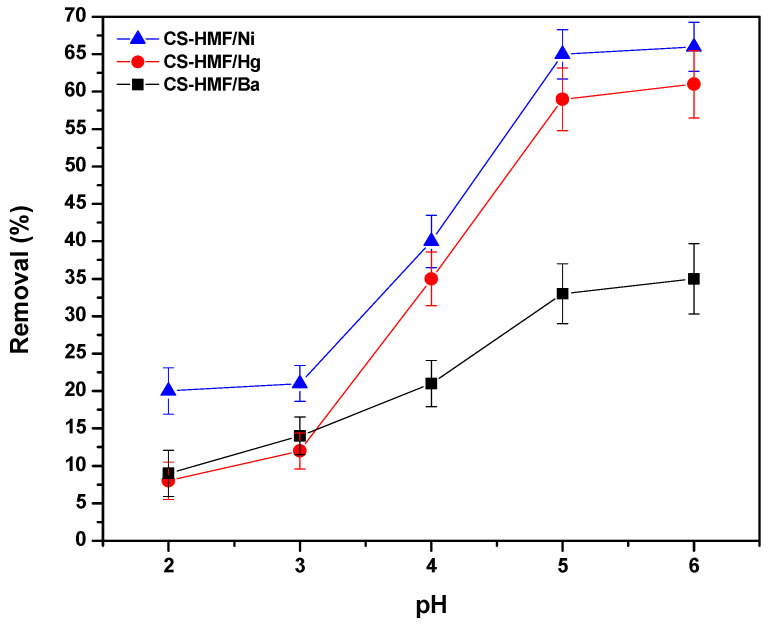
pH effect on the uptake of Ni^2+^, Hg^2+^, and Ba^2+^, from aqueous solutions (single-component) by using CS-HMF adsorbent.

During the process of removal of model pollutants, the pH effect plays a significant role because it controls the generated interactions (adsorbate–adsorbent) [52]. The pH-behavior on the process of adsorption is shown in Figure 2. It can be observed that the trend of the obtained pH curves is the same in the case of Ni^2+^ and Hg^2+^ adsorption (meaning that when increasing the pH, the ion removal increases), while in the case of Ba^2+^, it differentiates. The latter increase is milder for Ba^2+^ compared to other ions (Ni^2+^ and Hg^2+^). The differentiation of the barium ions removal may be attributed to the affinity (ionic radius of barium ion is larger than that of nickel and mercury ion). However, the highest removal percentage that found, for all cases of heavy metal ions (Ni^2+^, Hg^2+^, Ba^2+^), was at pH = 6. The low uptake level of metal ions at high acidic conditions is attributed to the excess of hydrogen concentration (H^+^) ions, which compete with metal ions for binding sites on CS-HMF [53]. Additionally, the protonation of amino groups to varying degrees at strong acidic conditions results in the decrease in available surface sites, for the uptake of metal ions [54]. It must be noted that other research observes that the highest percentage for Ni^2+^, Hg^2+^, and Ba^2+^ uptake is in pH range 4–6 [52,53,54,55].

In detail, in the case of low values of pH (high acidic), a major interaction may be favored: the electrostatic repulsion between amino chitosan groups (protonated), and metal ions with positive charge, and more specific with those that are not reacted in the presence of cross-linker, (Equation (8)).

(8) $$M^{2+}\overset{\mathrm{repulsion}}{\underset{\mathrm{high}\;\mathrm{acidic}\;\mathrm{pH}}{↔}}\mathrm{CS}-NH_{3}^{+}$$

According to the aforementioned consideration, in the case of low values pH, the chitosan groups (amino) were protonated (NH_3_^+^), resulting in a decrease in the available number of surface sites (binding process) for the removal of Ni^2+^, Hg^2+^, and Ba^2+^ (i.e., uncharged groups of NH_2_ for chelation). Consequently, Ni^2+^, Hg^2+^, and Ba^2+^ had to compete with the positive charge (H^+^) concentrations, in order to be bonded onto the surface sites of CS-HMF adsorbent. By increasing pH from high to less acidic aqueous solution (from 2.0 to 6.0), the crucial adsorption interaction was the process of chelation/complexation, between the hydroxyl or amino chitosan groups, and more specific with those that do not react with the cross-linker and metal ions with a positive charge (Equation (9)):

(9) $$M^{2+}\overset{\mathrm{chelation}}{\underset{\mathrm{low}\;\mathrm{acidic}\;\mathrm{pH}}{↔}}\mathrm{CS}-NH_{2}•••M^{2+}\mathrm{or}\;\mathrm{CS}-\mathrm{OH}•••M^{2+}$$

**Figure 3:**
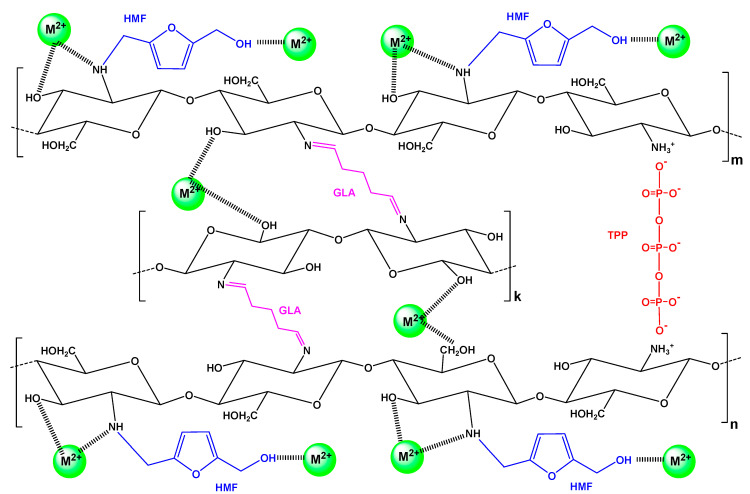
Proposed interactions for the removal of metal ions M^2+^ (Ni^2+^, Hg^2+^, Ba^2+^) by adsorption onto CS-HMF.

Additionally, it must be noted that according to BET analysis, CS-HMF was found to have a very limited surface area (5.31 m^2^/g), which is a typical non-porous material; the latter is confirmed in literature [56,57]. Therefore, the porosity does not play an important role on the adsorption interactions. A proposed mechanism based on the aforementioned is illustrated in Figure 3.

#### 3.2.2. Contact Time Effect

**Figure 4:**
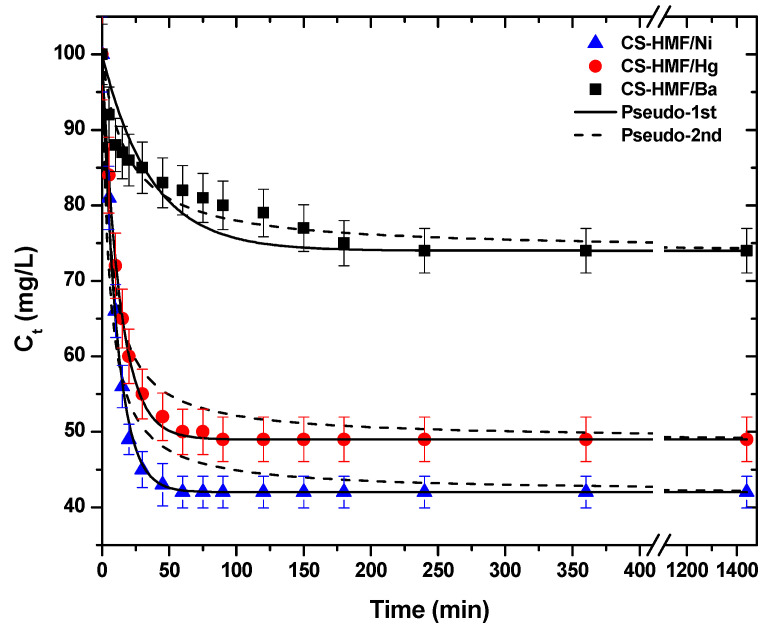
Kinetic data for the uptake of Ni^2+^, Hg^2+^, and Ba^2+^ from aqueous solutions (single-component). Fitting to pseudo-1st and pseudo-2nd order equations. Experimental conditions: T = 25 °C, C_0_ = 120.0 mg/L, N = 160 rpm, V = 20 mL, m = 0.02 g, pH = 6.

Another crucial factor for the process of adsorption is the effect of contact time. The kinetic trend is presented in Figure 4. As it can be observed, in the case of the removal of Ba^2+^ (single-component aqueous solution) from the liquid phase, the CS-HMF biomaterial has different kinetic behavior, when compared with the removal of Ni^2+^ and Hg^2+^. More specifically, the first time interval 0–250 min for the uptake of Ba^2+^ (1.42 Å) is gradual and then the equilibrium phase (plateau)appears. In the case of Ni^2+^ (0.83 Å), the time interval was shorter (0–60 min) until the equilibrium plateau was reached. For Hg^2+^ (1.33 Å) the time interval was 0–80 min until the equilibrium plateau was reached. The short time interval of Ni^2+^ and Hg^2+^ is accompanied by a sharp decrease in the concentration of model pollutants.

The experimental data from kinetic experiments were fitted to pseudo-1st and pseudo-2nd order equations. Table 1 presents the calculated parameters. It is clear that the correlation coefficients (R^2^) of pseudo-1st order are fitted better, in the case of Ni^2+^ (R^2^ = 0.997) and Hg^2+^ (R^2^ = 0.998) uptake, while in the case of Ba^2+^ removal, the best fitting was achieved with the pseudo-2nd order equation (R^2^ = 0.951). To better understand, in the case of PFO kinetics, if the concentration of one relative reagent remains constant (because it is supplied in great excess), its concentration can be absorbed at the expressed constant rate, obtaining the PFO reaction constant. If the diffusion film is rate-controlling, the rate of the adsorption will vary inversely with the particle size, the film thickness, and with the distribution coefficient. Therefore, the name “physisorption” was given, since the rate-limiting step in this kind of mechanism is diffusion and not dependent on the concentrations of both reactant (physical exchange). On the other hand, in the case of PSO, the chemical reaction seems significant in the rate-controlling step, the pseudo-second order chemical reaction kinetics provide the best correlation of the experimental data, and the adsorption’s mechanism is chemically rate-controlling (because of this it is called chemisorption). In this mechanism, the kinetics of sorption should correspond to a reversible second order reaction at low sorbate/sorbent ratios (first order at very low ratios) and two competitive reversible second order reactions at higher sorbate/sorbent ratios [47,58]. The aforementioned theoretical background can be used to explain the data of Table 1. The kinetic results showed that the adsorption of barium ions was slower (smaller rate constant than the adsorption of nickel and mercury ions. One possible explanation is the affinity (ionic radius). The same observation was found for both models fitted (PFO, PSO). Additionally, the adsorption kinetics of barium ions seems to better fit to the PFO than PSO model. One can correlate this finding to the sorption mechanism, suggesting that although the adsorption process is a combination of chemical and physical sorption, in this case, the physisorption probably dominates [47,58]. On the other hand, PSO is the best fitted model for barium ions, suggesting the strong chemical bonds of those ions with the CS-HMF.

#### 3.2.3. Effect of Initial Concentration/Temperature on Equilibrium

**Figure 5:**
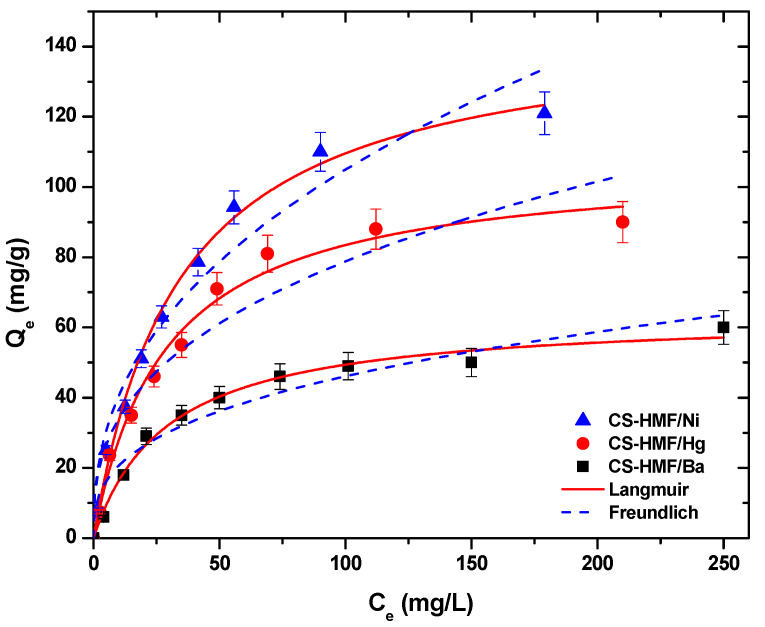
Equilibrium data for the uptake of Ni^2+^, Hg^2+^, and Ba^2+^ from aqueous solutions (single component). Fitting to the equations of Langmuir and Freundlich. Testing conditions: T = 25 °C, C_0_ = 10.0–300.0 (mg/L), N = 160 rpm, V = 20.0 mL, t = 200 min, m = 0.02 g, pH = 6.

Figure 5 presents the equilibrium isotherm curves, at T = 25 °C, after the fitting of Langmuir and Freundlich models for the uptake of Ni^2+^, Hg^2+^, and Ba^2+^ metal ions form aqueous solutions (single-component) by using CS-HMF adsorbent.

Table 2 demonstrates the fitting parameters calculated from the models, at T = 25 °C. The correlation coefficients (R^2^) from single-component aqueous solutions derived from the model of Langmuir were higher (0.991 ≤ R_L_^2^ ≤ 0.997) than those of Freundlich (0.908 ≤ R_F_^2^ ≤ 0.978), indicating that the model of Langmuir fits better to the experimental data (Figure 5). CS-HMF presents high adsorption abilities (T = 25 °C) for Ni^2+^, Hg^2+^, and Ba^2+^ with Q_m_ value equal to 147, 107, and 64 mg/g, respectively (Table 2).

**Figure 6:**
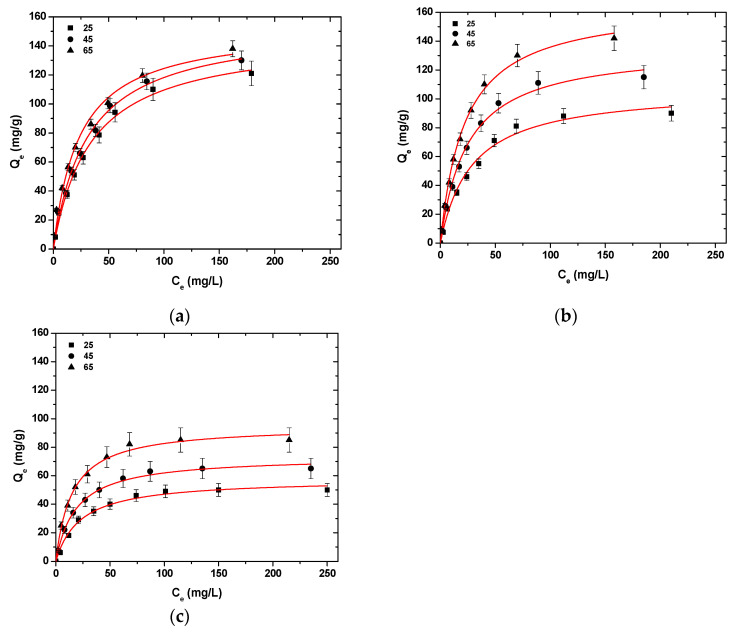
Isotherms for the uptake of (**a**) Ni^2+^; (**b**) Hg^2+^; (**c**) Ba^2+^ from aqueous solutions (single component). Testing conditions: T = 25–65 °C, C_0_ = 10.0–300.0 (mg/L), N = 160 rpm, V = 20.0 mL, t = 200 min, m = 0.02 g, pH = 6.

Figure 6 presents the isotherms curves after the fitting of Langmuir model (T = 25–65 °C). The Langmuir model was selected because, after the initial experiments, at T = 25 °C, it was determined that the model of Langmuir has a better fitting to the data obtained from the experimental process. In all cases of metal ion adsorption, with the temperature increase from 25 °C to 65 °C, an uptake increase in Ni^2+^, Hg^2+^, and Ba^2+^ was observed (Table 3).

More specifically, for Ni^2+^ uptake, CS-HMF enhanced its adsorption ability by 8 and 6 mg/g at temperatures 45 and 65 °C, while for Hg^2+^ uptake, the adsorption ability enhanced by 29 and 59 mg/g at temperatures 45 and 65 °C, respectively. Now, for Ba^2+^ uptake, the effect of the temperature increases the adsorption ability of CS-HMF derivative by 10 and 31 mg/g at temperatures 45 and 65 °C, respectively. Therefore, it can be concluded that with the temperature increase (25–65 °C), an enhancement of adsorption ability can be observed, at approximately 4.1, 55.2, and 48.5% for Ni^2+^, Hg^2+^, and Ba^2+^, respectively.

It seemed that the increase in temperature improved the diffusivity of heavy metal ions on water and augmented their adsorption. In order to state the above consideration, the values of Ni^2+^, Hg^2+^, and Ba^2+^ diffusivity in water (denoted as D_pw_) were calculated at three temperatures (25, 45, and 65 °C) according to the Wilke−Chang correlation [59] (Equations (10) and (11), respectively):

(10) $$D_{pw}=(7.4\times 10^{-12})\frac{T{\left(2.6M\right)}^{0.5}}{\eta V^{0.6}}$$

where V (cm^3^/mol) is the molar volume of ion; M (g/mol) is the molecular weight of the solvent (water); T (K) is temperature; η (cP) represents the dynamic viscosity of the solvent, and in our case, for water was found at three temperatures (25, 45, 65 °C) as follows [60], where A = 2.414 × 10^−2^ cP; B = 247.8 K; L = 140 K.

(11) $$\eta =10^{B/(T-L)}A$$

In the following table (Table 4), the diffusion coefficients were presented for the different temperatures. It seemed that the change in D_pw_ for Ni^2+^ was higher than that of Hg^2+^, and Ba^2+^, which explained the adsorption behavior in high temperatures. The above coefficients confirm that the effect of temperature was a key-factor in the process, affecting the diffusion of theses ion in the solution and making easier (more favorable) their contact with the adsorbent (CS-HMF).

##### Thermodynamic Estimation

Based on the equilibrium experiments (isotherms), the values of ΔG^0^, ΔH^0^, and ΔS^0^, were calculated at predefined temperatures (25, 45, and 65 °C), which correspond to Gibbs’ free energy change (kJ/mol), enthalpy (kJ/mol), and entropy (kJ/mol K), respectively. For the determination of thermodynamic parameters aforementioned, where C_s_ and R represent the quantity adsorbed on the surface of solid at the phase of equilibrium (mg/L) and universal gas constant (8.314 J/mol K), it will be used the following equation system [61].

(12) $$K_{c}\;=\;\frac{C_{s}}{C_{e}}$$

(13) $${\Delta G}^{0}\;=-R\;T\;\ln(K_{c})$$

(14) $${\Delta G}^{0}\;=\;\Delta H^{0}-\;T\;{\Delta S}^{0}$$

(15) $$\ln\left(K_{c}\right)\;=\;-\;\left(\frac{{\Delta H}^{0}}{R}\right)\frac{1}{T}+\frac{{\Delta S}^{0}}{R}$$

The value of ΔG^0^ was calculated from (Equation (13)), while ΔH^0^ and ΔS^0^ were estimated from the slop and intercept from the graph, which obtained between ln(K_c_) vs. 1/T. Table 5 depicts the resulting thermodynamic parameters, and more specifically, all parameters aforementioned (pH = 6) at specific initial concentrations of metal ions (10, 50, 120, and 200 mg/L) and also at predefined temperatures (25, 45, and 65 °C).

The increased trend of negative ΔG^0^ values, as for Ni^2+^ and Hg^2+^ uptakes, for temperatures 25, 45, and 65 °C at initial concentrations 10, 50, 120, and 200 mg/L, respectively, indicates that the process of uptake becomes spontaneous and feasible, when the temperature increases. In addition, the Ba^2+^ uptake, for temperatures 25, 45, and 65 °C at initial concentration 10 and 50 mg/L, respectively, indicates that the uptake process becomes spontaneous and feasible for the adsorption of metallic ions when the temperature increases. However, at initial concentrations 125 and 200 mg/L, the values of ΔG^0^ had a positive sign, indicating that the uptake process was not as favorable, as in other cases (negative values). Despite this, with the increase in temperature from 25 to 65 the values of ΔG^0^ decrease further, indicating a more favorable removal of Ba^2+^ with the temperature increase. In addition, Ni^2+^, Hg^2+^, and Ba^2+^ removals were evaluated as endothermic reactions, because all values of ΔH^0^ were positive. The positive ΔS^0^ values found reflect the sorbent affinity of CS-HMF adsorbent with the model pollutants (Ni^2+^, Hg^2+^, and Ba^2+^). However, the aforementioned positive values depict high randomness at the interface of solid/solution, during the metal ions sorption (Ni^2+^, Hg^2+^, and Ba^2+^) onto CS-HMF biobased adsorbent and also reveal that some changes happen in the structure of adsorbate and adsorbent. During the process of sorption, the adsorbed water-soluble molecules, which are displaced by the species of Ni^2+^, Hg^2+^, or Ba^2+^, earned more translational entropy than is lost, due to the presence of Ni^2+^, Hg^2+^, or Ba^2+^ species (adsorbate species/molecules), resulting in an increased randomness during the interaction of metal ions with CS-HMF derivative. The process of adsorption is not dominated by entropic but from enthalpic effects because |ΔH^0^_net_| > |T∙ΔS^0^|, indicating that the process of sorption was enthalpy-driven, for all cases of metal ions removal. Additionally, the positive value of ΔS^0^ indicated an increased freedom degree of the adsorbed solvent molecules.

### 3.3. Adsorption Evaluation—Mixtures

**Figure 7:**
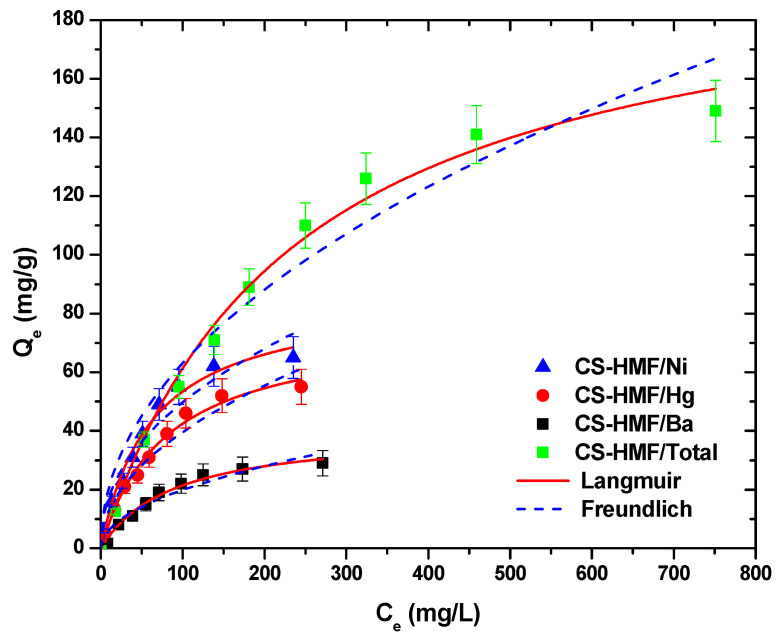
Equilibrium data for the uptake of Ni^2+^, Hg^2+^, and Ba^2+^ in multi-component aqueous solution, fitting to the equations of Langmuir and Freundlich. Testing conditions: T = 25 °C, C_0_ = 10.0–300.0 (mg/L), N = 160 rpm, V = 20.0 mL, t = 200 min, m = 0.02 g, pH = 6.

Figure 7 presents the isotherms of metal ion uptake in multi-component aqueous solution. The fitting was achieved both with Langmuir and Freundlich models (T = 25 °C).

**Figure 8:**
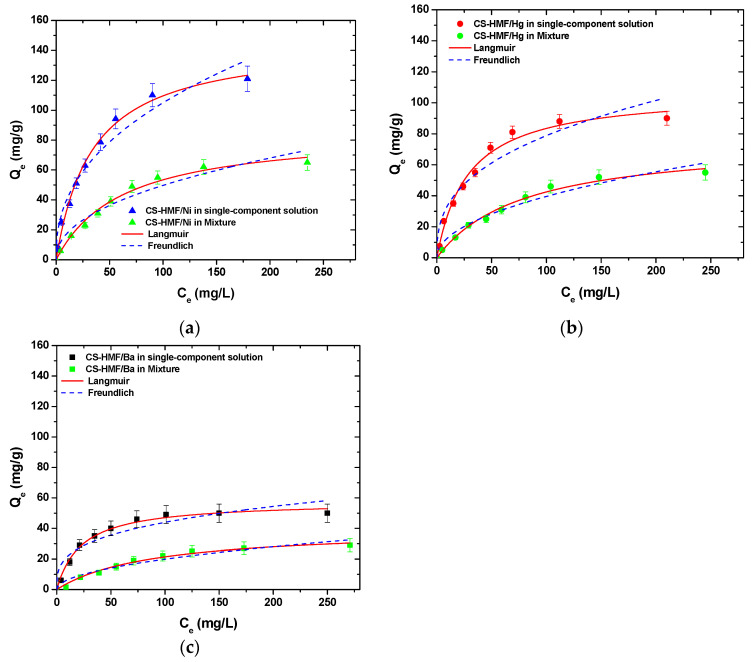
Equilibrium data for the uptake of (**a**) Ni^2+^; (**b**) Hg^2+^; (**c**) Ba^2+^ from single- and multi-component aqueous solutions, fitting to the equations of Langmuir and Freundlich. Testing conditions: T = 25 °C, C_0_ = 10.0–300.0 (mg/L), N = 160 rpm, V = 20.0 mL, t = 200 min, m = 0.02 g, pH = 6.

It is obvious that CS-HMF biomaterial can remove various types of metal ions from liquid phase simultaneously (in multi-component). The isothermal parameters were obtained after the experimental data fitting to the equations of Langmuir and Freundlich (Table 6). The experimental data for the multi-component aqueous solution were also applied to the models of Langmuir and Freundlich and compared with the initial parameters of equilibrium derived from single-component aqueous solutions, as presented in Figure 8. As it can be clearly seen from that figure, the co-existence of various metal ions in the mixture affected their removal. CS-HMF has 147 mg/g maximum adsorption capacity in single-component solutions, while in the presence of other two metal ions, the capacity is 40% lower (87 mg/g). In the case of mercury ions, the decrease is 30% (76 mg/g) and for barium ions is 35% (41 mg/g). The decrease in all ion capacities was in the range 30–40%, which means that the ions affinity in single-component solutions is kept in the case of mixture. Another important finding is that the total adsorption capacity found (meaning the sum of the metal ions capacities in the mixture) is slightly higher (204 mg/g) than in the case of single-component solutions (147, 107, and 64 mg/g). This again is in accordance with the affinity findings found before.

Table 6 presents the equilibrium parameters derived from Figure 8, and more specifically, the equilibrium parameters for Ni^2+^, Hg^2+^, and Ba^2+^ adsorption for single- and multi-component aqueous solutions onto CS-HMF biobased derivative (T = 25 °C) are presented.

The correlation coefficients (R^2^) from multi-component aqueous solution derived also from the models of Langmuir and Freundlich. For the Langmuir model, in the case of single-component aqueous solutions (Table 6), the correlation coefficients (R^2^) were (0.988 ≤ R_L_^2^ ≤ 0.994), while for multi-component aqueous solution, they were (0.987 ≤ R_L_^2^ ≤ 0.991). Now, in the case of Freundlich model for single-component aqueous solution, the correlation coefficients (R^2^) were (0.928 ≤ R_F_^2^ ≤ 0.959), while for multi-component aqueous solution, they were (0.943 ≤ R_F_^2^ ≤ 0.964). It is worth to note that in the cases of Ni^2+^ and Ba^2+^ metal ions removal, the correlation coefficients (R^2^) of Langmuir and Freundlich models were decreased for multi-component aqueous solution, while increased in the case of Hg^2+^ metal ions. However, if compare the aforementioned results with the single-component aqueous solutions results, a decrease in adsorption capacity of approximately 41, 29, and 36% for Ni^2+^, Hg^2+^, and Ba^2+^ metal ions, respectively, can be observed. In the case of total removal of Ni^2+^, Hg^2+^, and Ba^2+^ from multi-component aqueous solution, CS-HMF seems to have in total Q_m_ = 204 mg/g (Table 6), indicating that the CS-HMF derivative is a multifunctional adsorbent material.

### 3.4. Comparisons

To evaluate the effectiveness of CS-HMF adsorbent, a comparative table was carried out (Table 7). More specifically, the adsorption ability of various adsorbents (mainly CS) for the removal of Ni^2+^, Hg^2+^, and Ba^2+^ from single-component aqueous solutions is presented. It is obvious that the CS-HMF is a very effective adsorbent material, when compared with other studies, and especially for Ni^2+^ uptake.

In the case of multi-composite aqueous solution, there is not an extensive bibliography. In the study of Leos Doskocil and Miloslav Pekar, the removal of metal ions (Pb^2+^, Cu^2+^, Zn^2+^, and Cd^2+^) from multi-composite aqueous solution, using lignite was achieved, and the total adsorption capacity was found to be 85 mg/g [73]. Despite the relatively good adsorption capacity of lignite, it must be mentioned that it is not a renewable material and also is not abundant in nature. In another study from Kuang He et al. was achieved the removal of metal ions (Pb^2+^, Cd^2+^, Cu^2+^, Ni^2+^, and Mn^2+^) from multi-composite aqueous solution, using zeolite, and the total adsorption capacity was found to be 121.32 mg/g [74]. Despite that, zeolite can be found in nature, as in the case of lignite (not abundant), and also can be produced in industrial scale. The study of Abbas Afkhami et al. achieved the removal of metal ions (Pb^2+^, Cd^2+^, Cr^3+^, Co^2+^, Ni^2+^, and Mn^2+^) from multi-composite aqueous solution, using nano-alumina modified with 2,4-dinitrophenylhydrazine, and the total adsorption capacity was found to be 349.4 mg/g [75]. However, the total adsorption capacity of CS-HMF adsorbent for the removal of metal ions (Ni^2+^, Hg^2+^, Ba^2+^) from multi-component aqueous solution was found to be 204 mg/g, indicating that is a multifunctional adsorbent material. Finally, it can be supposed that if four or five model pollutants were used, as in the aforementioned studies, the adsorption capacity of CS-HMF biobased adsorbent will increase further, due to the presence of a different ionics radius in aqueous solution.

## 4. Conclusions

In this experimental study, chitosan grafted with 5-hydroxymethyl-furfural was used for the uptake of nickel, mercury, and barium metal ions from aqueous solutions. It can be concluded that the chitosan grafted with 5-hydroxymethyl-furfural can be used as biobased adsorbent material for the uptake of metal ions from single- and multi-component aqueous solutions, indicating its multi-functionality. Additionally, with the increase in temperature from 25 to 65 °C for single-component aqueous solutions, the adsorption capacity was increased at approximately 4.1, 55.2, and 48.5% for Ni_2+_, Hg^2+^, and Ba^2+^ metal ions, respectively. The correlation coefficients (R^2^) of pseudo-1st order are fitted better, in the case of Ni^2+^ (R^2^ = 0.997) and Hg^2+^ (R^2^ = 0.998) uptake, while in the case of Ba^2+^ removal, the pseudo-2nd order kinetic equation (R^2^ = 0.951) fits better. According to the thermodynamic studies, it was found that the enthalpy (ΔH^0^, kJ/mol) had positive values for the uptake of nickel, mercury, and barium metal ions by single-component aqueous solutions, indicating the endothermic nature of reactions during the process of adsorption. However, the positive values of entropy (ΔS^0^, kJ/mol K) for the uptake of nickel, mercury, and barium metal ions by single-component aqueous solutions indicated the sorbent affinity of adsorbent with the model pollutants. The increased trend of negative values of ΔG^0^, indicating that the process of adsorption becomes spontaneous and feasible, on metal ions sorption with the temperature increased. Finally, it was concluded that the correlation coefficients (R^2^) of the Langmuir model fit better to the experimental data than that of the Freundlich model for single- and multi-component aqueous solutions, indicating monolayer coverage of Ni^2+^, Hg^2+^, and Ba^2+^ onto the surface of CS-HMF.

## Figures and Tables

**Table 1:** Pseudo-1st and pseudo-2nd order equations for the uptake of Ni^2+^, Hg^2+^, and Ba^2+^ from aqueous solutions (single component).

	Pseudo-1st Order		Pseudo-2nd Order	
	k_1_	R^2^	k_2_	R^2^
Adsorbent	(min^−1^)		(g mg^−1^ min^−1^)	
CS-HMF/Ni	0.09184	0.997	0.19013	0.943
CS-HMF/Hg	0.07621	0.998	0.15267	0.963
CS-HMF/Ba	0.02768	0.817	0.05541	0.951

**Table 2:** Equilibrium parameters for Ni^2+^, Hg^2+^, and Ba^2+^ adsorption at 25 °C (single-component aqueous solutions) onto CS-HMF bioadsorbent.

Model	Equation	Parameter	Ni^2^	Hg^2^	Ba^2+^
Langmuir	$Q_{e}=\frac{Q_{m}K_{L}C_{e}}{1+K_{L}C_{e}}$	Q_m_ (mg/g)	147	107	64
		K_L_ (L/mg)	0.029	0.035	0.034
		R^2^	0.994	0.988	0.991
Freundlich	$Q_{e}=K_{F}C_{e}{}^{1/n}$	K_F_ (mg^1 − 1/n^ L^1/n^ g^−1^)	15.549	14.618	9.22
		n (−)	2.411	2.733	2.864
		R^2^ (−)	0.959	0.928	0.95

**Table 3:** Equilibrium parameters for Ni^2+^, Hg^2+^, and Ba^2+^ adsorption at 25, 45, and 65 °C (single-component aqueous solutions) onto CS-HMF bioadsorbent.

		Ni^2^			Hg^2^			Ba^2+^		
Model	T (°C)	25	45	65	25	45	65	25	45	65
Langmuir	Q_m_ (mg/g)	147	155	153	107	136	166	64	74	95
	K_L_ (L/mg)	0.029	0.032	0.043	0.035	0.040	0.045	0.034	0.053	0.066
	R^2^	0.994	0.994	0.991	0.988	0.994	0.997	0.991	0.994	0.994

**Table 4:** Diffusion parameters of ions in water at (25, 45, and 65 °C).

	D_pw_ (m^2^/s)		
Ion	25	45	65
Ni^2+^	5.45 × 10^−9^	8.72 × 10^−9^	12.52 × 10^−9^
Hg^2+^	3.45 × 10^−9^	5.53 × 10^−9^	8.12 × 10^−9^
Ba^2+^	1.89 × 10^−9^	3.04 × 10^−9^	4.47 × 10^−9^

**Table 5:** Thermodynamic parameters for the uptake of Ni^2+^, Hg^2+^, and Ba^2+^ from aqueous solutions (single component).

	C_0_	T	K_c_	ΔG^0^	ΔH^0^	ΔS^0^
	(mg/L)	(K)		(kJ/mol)	(kJ/mol)	(kJ/mol K)
CS-HMF/Ni	10	298	4.56	−3.76		
		318	5.67	−4.59	14.13	0.060
		338	9	−6.18		
	50	298	2.97	−2.70		
		318	3.35	−3.19	11.78	0.048
		338	5.25	−4.66		
	120	298	1.90	−1.59		
		318	2.14	−2.01	5.98	0.025
		338	2.53	−2.61		
	200	298	1.22	−0.50		
		318	1.37	−0.83	4.07	0.015
		338	1.48	−1.11		
CS-HMF/Hg	10	298	2.85	−2.59		
		318	5.67	−4.59	26.55	0.098
		338	10.11	−6.50		
	50	298	2.33	−2.10		
		318	3.55	−3.35	16.97	0.064
		338	5.25	−4.66		
	120	298	1.45	−0.92		
		318	2.24	−2.14	17.14	0.061
		338	3.29	−3.34		
	200	298	0.79	0.60		
		318	1.25	−0.58	18.01	0.058
		338	1.86	−1.74		
CS-HMF/Ba	10	298	1.50	−1.00		
		318	2.33	−2.24	20.74	0.072
		338	4.00	−3.90		
	50	298	1.38	−0.80		
		318	2.13	−1.99	19.68	0.069
		338	3.55	−3.56		
	120	298	0.62	1.18		
		318	0.94	0.18	19.11	0.060
		338	1.55	−1.24		
	200	298	0.33	2.72		
		318	0.48	1.93	16.62	0.047
		338	0.74	0.85		

**Table 6:** Equilibrium parameters for Ni^2+^, Hg^2+^, and Ba^2+^ adsorption (single- and multi-component aqueous solutions), onto CS-HMF adsorbent.

		Ni^2^		Hg^2^		Ba^2+^		
Model	Parameters	Single	Mix	Single	Mix	Single	Mix	Total
Langmuir	Q_m_ (mg/g)	147	87	107	76	64	41	204
	K_L_ (L/mg)	0.029	0.016	0.035	0.012	0.034	0.011	0.004
	R^2^	0.994	0.989	0.988	0.991	0.991	0.987	0.992
Freundlich	K_F_ (mg^1 − 1/n^ L^1/n^ g^−1^)	15.549	6.194	14.618	4.221	9.22	2.053	6.827
	n	2.411	2.211	2.733	2.056	2.864	2.027	2.071
	R^2^	0.959	0.948	0.928	0.964	0.95	0.943	0.954

**Table 7:** Comparative study for the uptake of Ni^2+^, Hg^2+^, and Ba^2+^, from single-component aqueous solutions, using various adsorbents (mainly CS). Below are presented the adsorption capacities according to the Langmuir model (T = 25 °C).

Adsorbent	Metal Ion	Q_m_(mg/g)	Reference
Porous thiourea-grafted-CS hydrogels	Ni^2+^	132.5	[62]
CS coated polyvinyl chloride beads		120.5	[53]
Magnetic activated carbon/CS beads		108.7	[63]
DTPA-modified CS/polyethylene oxide nanofibers		56	[64]
CS/magnetite composite beads		52.55	[12]
Modified magnetic CS chelating resin		40.15	[7]
Chemically cross-linked metal complexed CS		37.88	[65]
Ethylenediaminetetraacetic acid-CS		24.35	[66]
Diethylenetriaminepentaacetic acid -CS		24.16	[66]
Thiourea-modified magnetic CS microspheres		15.3	[16]
CS-MAA nanoparticles		0.87	[67]
CS-HMF		147	This study
Cross-linked magnetic CS-phenylthiourea resin	Hg^2+^	135	[68]
Formaldehyde modified CS-thioglyceraldehyde		98	[69]
Thiocarbohydrazide-chitosan		52.63	[70]
CS		24	[71]
CS-HMF		107	This study
Weathered basalt/CS	Ba^2+^	45.78	[52]
Dolomite powder		3.96	[72]
Perlite		2.49	[13]
CS-HMF		64	This study

## Data Availability

The data presented in this study are available upon request from the corresponding author.

## References

[1] Ali A., Ing A.W.C., Abdullah W.R.W., Hamzah S., Azaman F. (2020). Preparation of High-Performance Adsorbent from Low-Cost Agricultural Waste (Peanut Husk) Using Full Factorial Design: Application to Dye Removal. Biointerface Res. Appl. Chem..

[2] Ali S.F.A., Gad E.S. (2020). Investigation of an adsorbent based on novel starch/chitosan nanocomposite in extraction of indigo carmine dye from aqueous solutions. Biointerface Res. Appl. Chem..

[3] Batool M., Khurshid S., Qureshi Z.M., Daoush W.M., Hashmi F., Mehboob N. (2020). Effective adsorptive removal of azodyes on synthesized copper oxide nanoparticles. Biointerface Res. Appl. Chem..

[4] Cotrim A.C.M., França E.L., França A.C.H., Martins J.S., Silva K.P.G., Ghalfi Y.C., Machado I.T., Tozetti I.A. (2020). Effect of polyethylene glycol microspheres adsorbed with melatonin on oxidative stress and viscosity of cervical mucus samples infected with human papillomavirus. Biointerface Res. Appl. Chem..

[5] Kulkarni S. (2020). Synthesis, characterization and performance of low-cost unconventional adsorbents derived from waste materials. Biointerface Res. Appl. Chem..

[6] Crini G. (2005). Recent developments in polysaccharide-based materials used as adsorbents in wastewater treatment. Prog. Polym. Sci..

[7] Monier M., Ayad D., Wei Y., Sarhan A. (2010). Adsorption of Cu(II), Co(II), and Ni(II) ions by modified magnetic chitosan chelating resin. J. Hazard. Mater..

[8] Wadi V.S., Mittal H., Fosso-Kankeu E., Jena K.K., Alhassan S.M. (2020). Mercury removal by porous sulfur copolymers: Adsorption isotherm and kinetics studies. Colloids Surf. A: Physicochem. Eng. Asp..

[9] Zhang D., Crini G., Lichtfouse E., Rhimi B., Wang C. (2020). Removal of Mercury Ions from Aqueous Solutions by Crosslinked Chitosan-based Adsorbents: A Mini Review. Chem. Rec..

[10] Farooq U., Kozinski J.A., Khan M.A., Athar M. (2010). Biosorption of heavy metal ions using wheat based biosorbents—A review of the recent literature. Bioresour. Technol..

[11] Vijaya Y., Popuri S.R., Boddu V.M., Krishnaiah A. (2008). Modified chitosan and calcium alginate biopolymer sorbents for removal of nickel (II) through adsorption. Carbohydr. Polym..

[12] Tran H.V., Tran L.D., Nguyen T.N. (2010). Preparation of chitosan/magnetite composite beads and their application for removal of Pb(II) and Ni(II) from aqueous solution. Mater. Sci. Eng. C.

[13] Torab-Mostaedi M., Ghaemi A., Ghassabzadeh H., Ghannadi-Maragheh M. (2011). Removal of strontium and barium from aqueous solutions by adsorption onto expanded perlite. Can. J. Chem. Eng..

[14] Abdulkhair B., Salih M., Modwi A., Adam F., Elamin N., Seydou M., Rahali S. (2021). Adsorption behavior of barium ions onto ZnO surfaces: Experiments associated with DFT calculations. J. Mol. Struct..

[15] Baldermann A., Grießbacher A.C., Baldermann C., Purgstaller B., Letofsky-Papst I., Kaufhold S., Dietzel M. (2018). Removal of Barium, Cobalt, Strontium, and Zinc from Solution by Natural and Synthetic Allophane Adsorbents. Geoscience.

[16] Zhou L., Wang Y., Liu Z., Huang Q. (2009). Characteristics of equilibrium, kinetics studies for adsorption of Hg(II), Cu(II), and Ni(II) ions by thiourea-modified magnetic chitosan microspheres. J. Hazard. Mater..

[17] Dąbrowski A., Hubicki Z., Podkościelny P., Robens E. (2004). Selective removal of the heavy metal ions from waters and industrial wastewaters by ion-exchange method. Chemosphere.

[18] Blanchard G., Maunaye M., Martin G. (1984). Removal of heavy metals from waters by means of natural zeolites. Water Res..

[19] Chen S., Zhao W. (2019). Adsorption of Pb2+ from Aqueous Solutions Using Novel Functionalized Corncobs via Atom Transfer Radical Polymerization. Polymers.

[20] Pham T.D., Vu T.N., Nguyen H.L., Le P.H.P., Hoang T.S. (2020). Adsorptive Removal of Antibiotic Ciprofloxacin from Aqueous Solution Using Protein-Modified Nanosilica. Polymers.

[21] Ahmad A., Jamil S.N.A.M., Choong T.S., Abdullah A.H., Mastuli M.S., Othman N., Jiman N. (2019). Green Flexible Polyurethane Foam as a Potent Support for Fe-Si Adsorbent. Polymers.

[22] Maponya T.C., Ramohlola K.E., Kera N.H., Modibane K.D., Maity A., Katata-Seru L., Hato M.J. (2020). Influence of Magnetic Nanoparticles on Modified Polypyrrole/m-Phenylediamine for Adsorption of Cr(VI) from Aqueous Solution. Polymers.

[23] Ren L., Yang Z., Huang L., He Y., Wang H., Zhang L. (2020). Macroscopic Poly Schiff Base-Coated Bacteria Cellulose with High Adsorption Performance. Polymers.

[24] Shaipulizan N.S., Jamil S.N.A.M., Kamaruzaman S., Subri N.N.S., Adeyi A.A., Abdullah A.H., Abdullah L.C. (2020). Preparation of Ethylene Glycol Dimethacrylate (EGDMA)-Based Terpolymer as Potential Sorbents for Pharmaceuticals Adsorption. Polymers.

[25] Khan M.A., Momina, Siddiqui M.R., Otero M., Alshareef S.A., Rafatullah M. (2020). Removal of Rhodamine B from Water Using a Solvent Impregnated Polymeric Dowex 5WX8 Resin: Statistical Optimization and Batch Adsorption Studies. Polymers.

[26] Sudre G., Siband E., Gallas B., Cousin F., Hourdet D., Tran Y. (2020). Responsive Adsorption of N-Isopropylacrylamide Based Copolymers on Polymer Brushes. Polymers.

[27] Zhang W., Yang Z.-Y., Cheng X.-W., Chen G., Qiao Y.-F. (2019). Adsorption, Antibacterial and Antioxidant Properties of Tannic Acid on Silk Fiber. Polymers.

[28] Guo W., Xia T., Pei M., Du Y., Wang L. (2019). Bentonite Modified by Allylamine Polymer for Adsorption of Amido Black 10B. Polymers.

[29] Kim S.W., Sohn J.S., Kim H.K., Ryu Y., Cha S.W. (2019). Effects of Gas Adsorption on the Mechanical Properties of Amorphous Polymer. Polymers.

[30] Wang C., Zhao J., Wang S., Zhang L., Zhang B. (2019). Efficient and Selective Adsorption of Gold Ions from Wastewater with Polyaniline Modified by Trimethyl Phosphate: Adsorption Mechanism and Application. Polymers.

[31] Huang W., Diao K., Tan X., Lei F., Jiang J., Goodman B.A., Ma Y., Liu S. (2019). Mechanisms of Adsorption of Heavy Metal Cations from Waters by an Amino Bio-Based Resin Derived from Rosin. Polymers.

[32] Kong W.-Q., Chang M., Zhang C., Liu X., He B., Ren J. (2019). Preparation of Xylan-g-/P(AA-co-AM)/GO Nanocomposite Hydrogel and its Adsorption for Heavy Metal Ions. Polymers.

[33] Sims R.A., Harmer S.L., Quinton J.S. (2019). The Role of Physisorption and Chemisorption in the Oscillatory Adsorption of Organosilanes on Aluminium Oxide. Polymers.

[34] Othman N.A.F., Selambakkannu S., Abdullah T.A.T., Hoshina H., Sattayaporn S., Seko N. (2019). Selectivity of Copper by Amine-Based Ion Recognition Polymer Adsorbent with Different Aliphatic Amines. Polymers.

[35] Acharya R., Parida K. (2020). A review on adsorptive remediation of Cr(Vi) by magnetic iron oxides and their modified forms. Biointerface Res. Appl. Chem..

[36] Anuar F.I., Hadibarata T., Syafrudin M., Fona Z. (2020). Removal of procion red MX-5B from aqueous solution by adsorption on parkia speciosa (Stink bean) peel powder. Biointerface Res. Appl. Chem..

[37] Kanthasamy S., Hadibarata T., Hidayat T., Alamri S.A., Al-Ghamdi A.A. (2020). Adsorption of azo and anthraquinone dye by using watermelon peel powder and corn peel powder: Equilibrium and kinetic studies. Biointerface Res. Appl. Chem..

[38] Lau K.B.K., Hadibarata T., Elwina E., Dewi R., Alsahli A.A., Alaraidh I.A., Al-Ghamdi A.A. (2020). Reactive dyes adsorption via citrus hystrix peel powder and zea mays cob powder: Characterization, isotherm and kinetic studies. Biointerface Res. App. Chem..

[39] Satti Z., Akhtar M., Mazhar N., Khan S.U., Ahmed N., Yasir Q.M., Irshad M., Pervaiz R., Ahmad W. (2020). Adsorption of cadmium from aqueous solution onto untreated gypsum rock material: Equilibrium and kinetics. Biointerface Res. Appl. Chem..

[40] Ngah W.W., Teong L., Hanafiah M. (2011). Adsorption of dyes and heavy metal ions by chitosan composites: A review. Carbohydr. Polym..

[41] Asandei D., Bulgariu L., Bobu E. (2009). Lead (II) removal from aqueous solutions by adsorption onto chitosan. Cellul. Chem. Technol..

[42] Liakos E.V., Mitkidou S.A., Mitropoulos A.C., Kyzas G.Z. (2018). Nanohybrid chitosans in sorption technology. Composite Nanoadsorbents.

[43] Mone M., Lambropoulou D.A., Bikiaris D.N., Kyzas G. (2020). Chitosan grafted with biobased 5-hydroxymethyl-furfural as adsorbent for copper and cadmium ions removal. Polymers.

[44] Siafaka P.I., Mone M., Koliakou I.G., Kyzas G.Z., Bikiaris D. (2016). Synthesis and physicochemical properties of a new biocompatible chitosan grafted with 5-hydroxymethylfurfural. J. Mol. Liq..

[45] Elouear Z., Bouzid J., Boujelben N., Feki M., Jamoussi F., Montiel A. (2008). Heavy metal removal from aqueous solutions by activated phosphate rock. J. Hazard. Mater..

[46] Lagergren S. (1898). About the theory of so-called adsorption of soluble substances. Handlingar.

[47] Ho Y.-S., McKay G. (1999). Pseudo-second order model for sorption processes. Process. Biochem..

[48] Ho Y.-S. (2006). Review of second-order models for adsorption systems. J. Hazard. Mater..

[49] Ho Y.-S. (2006). Second-order kinetic model for the sorption of cadmium onto tree fern: A comparison of linear and non-linear methods. Water Res..

[50] Langmuir I. (1918). The adsorption of gases on plane surfaces of glass, mica and platinum. J. Am. Chem. Soc..

[51] Freundlich H.F. (1906). Over the adsorption in solution. J. Physicochem..

[52] Mohamed E.A., Mobarak M., Kumar R., Barakat M., Bonilla-Petriciolet A., Seliem M.K., Selim A.Q. (2020). Novel hybrid multifunctional composite of chitosan and altered basalt for barium adsorption: Experimental and theoretical studies. Colloids Surf. A Physicochem. Eng. Asp..

[53] Popuri S.R., Vijaya Y., Boddu V.M., Krishnaiah A. (2009). Adsorptive removal of copper and nickel ions from water using chitosan coated PVC beads. Bioresour. Technol..

[54] Shafaei A., Ashtiani F.Z., Kaghazchi T. (2007). Equilibrium studies of the sorption of Hg(II) ions onto chitosan. Chem. Eng. J..

[55] Li N., Bai R., Liu C. (2005). Enhanced and Selective Adsorption of Mercury Ions on Chitosan Beads Grafted with Polyacrylamide via Surface-Initiated Atom Transfer Radical Polymerization. Langmuir.

[56] Stanley-Wood N., Chatterjee A. (1974). The comparison of the surface area of porous and non-porous solids as determined by diffusional flow and nitrogen adsorption. Powder Technol..

[57] Rinaudo M. (2006). Chitin and chitosan: Properties and applications. Prog. Polym. Sci..

[58] Ho Y.-S., Ofomaja E.A. (2006). Pseudo-second-order model for lead ion sorption from aqueous solutions onto palm kernel fiber. J. Hazard. Mater..

[59] Wilke C.R., Chang P. (1955). Correlation of diffusion coefficients in dilute solutions. AIChE J..

[60] Daugherty R.L., Franzini J.B. (1977). Fluid Mechanics with Engineering Applications.

[61] Kyzas G.Z., Lazaridis N.K., Mitropoulos A.C. (2012). Removal of dyes from aqueous solutions with untreated coffee residues as potential low-cost adsorbents: Equilibrium, reuse and thermodynamic approach. Chem. Eng. J..

[62] Ghiorghita C.-A., Borchert K.B., Vasiliu A.-L., Zaharia M.-M., Schwarz D., Mihai M. (2020). Porous thiourea-grafted-chitosan hydrogels: Synthesis and sorption of toxic metal ions from contaminated waters. Colloids Surfaces A Physicochem. Eng. Asp..

[63] Le V.T., Dao M.U., Le H.S., Tran D.L., Doan V.D., Nguyen H.T. (2020). Adsorption of Ni(II) ions by magnetic activated carbon/chitosan beads prepared from spent coffee grounds, shrimp shells and green tea extract. Environ. Technol..

[64] Surgutskaia N.S., Di Martino A., Zednik J., Ozaltin K., Lovecká L., Bergerová E.D., Kimmer D., Svoboda J., Sedlarik V. (2020). Efficient Cu2+, Pb2+ and Ni2+ ion removal from wastewater using electrospun DTPA-modified chitosan/polyethylene oxide nanofibers. Sep. Purif. Technol..

[65] Chen A.-H., Yang C.-Y., Chen C.-Y. (2009). The chemically crosslinked metal-complexed chitosans for comparative adsorptions of Cu(II), Zn(II), Ni(II) and Pb(II) ions in aqueous medium. J. Hazard. Mater..

[66] Repo E., Warchol J.K., Kurniawan T.A., Sillanpää M.E. (2010). Adsorption of Co(II) and Ni(II) by EDTA- and/or DTPA-modified chitosan: Kinetic and equilibrium modeling. Chem. Eng. J..

[67] Heidari A., Younesi H., Mehraban Z., Heikkinen H. (2013). Selective adsorption of Pb(II), Cd(II), and Ni(II) ions from aqueous solution using chitosan–MAA nanoparticles. Int. J. Biol. Macromol..

[68] Monier M., Abdel-Latif D. (2012). Preparation of cross-linked magnetic chitosan-phenylthiourea resin for adsorption of Hg(II), Cd(II) and Zn(II) ions from aqueous solutions. J. Hazard. Mater..

[69] Monier M. (2012). Adsorption of Hg2+, Cu2+ and Zn2+ ions from aqueous solution using formaldehyde cross-linked modified chitosan–thioglyceraldehyde Schiff’s base. Int. J. Biol. Macromol..

[70] Ahmad M., Manzoor K., Chaudhuri R.R., Ikram S. (2017). Thiocarbohydrazide Cross-Linked Oxidized Chitosan and Poly(vinyl alcohol): A Green Framework as Efficient Cu(II), Pb(II), and Hg(II) Adsorbent. J. Chem. Eng. Data.

[71] Miretzky P., Cirelli A.F. (2009). Hg(II) removal from water by chitosan and chitosan derivatives: A review. J. Hazard. Mater..

[72] Ghaemi A., Torab-Mostaedi M., Ghannadi-Maragheh M. (2011). Characterizations of strontium(II) and barium(II) adsorption from aqueous solutions using dolomite powder. J. Hazard. Mater..

[73] Doskočil L., Pekař M. (2012). Removal of metal ions from multi-component mixture using natural lignite. Fuel Process. Technol..

[74] He K., Chen Y., Tang Z., Hu Y. (2015). Removal of heavy metal ions from aqueous solution by zeolite synthesized from fly ash. Environ. Sci. Pollut. Res..

[75] Afkhami A., Saber-Tehrani M., Bagheri H. (2010). Simultaneous removal of heavy-metal ions in wastewater samples using nano-alumina modified with 2,4-dinitrophenylhydrazine. J. Hazard. Mater..

